# Downregulation of miR-151-5p Contributes to Increased Susceptibility to Arrhythmogenesis during Myocardial Infarction with Estrogen Deprivation

**DOI:** 10.1371/journal.pone.0072985

**Published:** 2013-09-09

**Authors:** Ying Zhang, Renjun Wang, Weijie Du, Shuxuan Wang, Lei Yang, Zhenwei Pan, Xuelian Li, Xuehui Xiong, Hua He, Yongfang Shi, Xue Liu, Shaonan Yu, Zhengang Bi, Yanjie Lu, Hongli Shan

**Affiliations:** 1 Department of Pharmacology (State-Province Key Laboratories of Biomedicine- Pharmaceutics of China, Key Laboratory of Cardiovascular Research, Ministry of Education), Harbin Medical University, Harbin, China; 2 Department of Orthopedics, the First Affiliated Hospital of Harbin Medical University, Harbin, China; University of Western Ontario, Canada

## Abstract

Estrogen deficiency is associated with increased incidence of cardiovascular diseases. But merely estrogen supplementary treatment can induce many severe complications such as breast cancer. The present study was designed to elucidate molecular mechanisms underlying increased susceptibility of arrhythmogenesis during myocardial infarction with estrogen deprivation, which provides us a new target to cure cardiac disease accompanied with estrogen deprivation. We successfully established a rat model of myocardial ischemia (MI) accompanied with estrogen deprivation by coronary artery ligation and ovariectomy (OVX). Vulnerability and mortality of ventricular arrhythmias increased in estrogen deficiency rats compared to non estrogen deficiency rats when suffered MI, which was associated with down-regulation of microRNA-151-5p (miR-151-5p). Luciferase Reporter Assay demonstrated that miR-151-5p can bind to the 3′-UTR of *FXYD1* (coding gene of phospholemman, PLM) and inhibit its expression. We found that the expression of PLM was increased in (OVX+MI) group compared with MI group. More changes such as down-regulation of Kir2.1/I_K1_, calcium overload had emerged in (OVX+MI) group compared to MI group merely. Transfection of miR-151-5p into primary cultured myocytes decreased PLM levels and [Ca^2+^]_i_, however, increased Kir2.1 levels. These effects were abolished by the antisense oligonucleotides against miR-151-5p. Co-immunoprecipitation and immunofluorescent experiments confirmed the co-localization between Kir2.1 and PLM in rat ventricular tissue. We conclude that the increased ventricular arrhythmias vulnerability in response to acute myocardial ischemia in rat is critically dependent upon down-regulation of miR-151-5p. These findings support the proposal that miR-151-5p could be a potential therapeutic target for the prevention of ischemic arrhythmias in the subjects with estrogen deficiency.

## Introduction

Women are not prone to develop cardiovascular disease before menopausal, but this advantage will disappear after menopause, suggesting that estrogen has a cardioprotective effect [1], [2]. Indeed, postmenopausal women are more prone to myocardial injuries in patients with myocardial ischemia (MI). Many observational studies have shown that postmenopausal women who receive estrogen replacement therapy have a lower rate of coronary heart disease (CHD) and cardiac death than those not receiving estrogen replacement therapy [3], [4], [5]. For years, the mechanism of this protective effect is due to inhibition of atherosclerosis [6]. But it can’t explain why susceptibility of arrhythmia increased before atherosclerotic plaque formation. Moreover, although estrogen replacement therapy can decrease the incidence of coronary heart disease, it can induce many severe complications such as breast cancer. So we explore new target to prevent and cure ischemia heart disease in postmenopausal women.

More than 5000 microRNAs (miRNAs), the single-stranded non-coding RNAs of approximately 22 nucleotides long, have been identified in humans [7]. They regulate gene expression by binding to the 3′-untranslated regions (UTRs) of target mRNAs through inexact sequence matching [8]. It has been identified that miRNAs had emerged as a critical node in posttranscriptional regulation of growth, apoptosis, and other cellular processes and as one of the most important regulators in human physiology and pathology [9]. In our study, we found that the level of miR-151-5p decreased significantly in myocardial ischemia. Whether this low level of miR-151-5p participates in the increasing susceptibility of arrhythmia when estrogen is deprived? So in this article we elucidate molecular and electrophysiologic mechanisms underlying increased susceptibility of myocardial ischemia-induced arrhythmias in rats with estrogen-deficiency.

## Materials and Methods

### Animals

Female Sprague-Dawley rats which weighed between 250g–300g each were used in the present study. This study was specifically approved by the ethic committees of Harbin Medical University. Use of animals was in accordance with the regulations of the ethic committees of Harbin Medical University, and confirmed with the *Guide for the Care and Use of Laboratory Animals* published by the US National Institutes of Health (NIH Publication No. 85-23, revised 1996).

### Rat Model of Estrogen-deficiency and Myocardial Ischemia (MI)

Rats were randomly divided into four groups: control (Ctl; sham-operated), MI (myocardial ischemia model with coronary artery ligation), OVX (estrogen deficiency with ovariectomy), and OVX+MI. All rats were anesthetized under intraperitoneal (i.p.) sodium pentobarbital (40 mg/kg). The standard electrocardiographic lead II were continuously recorded for a period of 0.5 h prior to surgery, then the rats were bilateral ovariectomized to establish estrogen-deficiency or sham-operated at 40 weeks of age. After the surgical procedure, the rats were given antibiotics, standard food and water feeding and housed in separate cages in the laboratory, which was kept at an ambient temperature of 20°C with a 12 h light/dark cycle. Myocardial ischemia model was established after estrogen-deficiency for 24 weeks. The MI or OVX+MI groups were anesthetized with sodium pentobarbital (40 mg/kg i.p.) then underwent ligation of the left anterior descending (LAD) coronary artery, as described previously [10].

### Scoring of Arrhythmia

Continuous standard electrocardiographic lead II recording was acquired for 1 hour after MI surgery. Arrhythmia scoring was as described by Curtis and Walker (1988) [11] as follows: 0 = no arrhythmia; 1≤10 s premature ventricular contraction (PVC) and/or ventricular tachycardia (VT); 2 = 11–30 s PVC and/or VT; 3 = 31–90 s PVC and/or VT; 4 = 91–180 s PVC and/or VT, or reversible ventricular fibrillation (VF) of <10 s; 5≥180 s PVC and/or VT, >10 s reversible VF, 6 = irreversible VF. Incidence of VF was calculated as percentage of animals with VF over the total number of animals studied. The incidence of arrhythmias and the survival rate were registered and evaluated, and the criteria of arrhythmia was similar to that described previously [10], [12].

### Measurements of Hemodynamic Function

Subsequently after ECG recordings, left ventricular end diastolic pressure (LVEDP) and time derivatives of pressure were recorded, and cardiac contraction (±dP/dt) and relaxation (±dP/dt) were measured and displayed on a polygraph [10], [13].

### Isolation of Ventricular Myocytes

Single ventricular myocytes were isolated using an established enzymatic digestion protocol from the rat hearts of different groups, as detailed previously [12], [14], [15], [16]. Rats were anaesthetized with sodium pentobarbital. The hearts were then rapidly excised and perfused according to the Langendorff method at a perfusion pressure of 60 mmHg with Ca^2+^-containing Tyrode solution containing (in mM): NaCl 137, KCl 5.4, CaCl_2_ 1.8, MgCl_2_ 1.0, HEPES 10, NaHCO_3_ 11.9, NaH_2_PO_4_ 0.33, glucose 10 (pH 7.4), then switched to Ca^2+^-free solution, followed by perfusion with the same Ca^2+^-free solution containing 0.05% collagenase and 0.1% bovine serum albumin. Ventricular tissues of peri-ischemic zone were minced into pieces, and stored in Kraftbruhe solution containing (in mM): glutamic acid 70, taurine 15, KCl 30, KH_2_PO_4_ 10, HEPES 10, MgCl_2_ 0.5, glucose 10 and EGTA 0.5; adjusted to pH 7.4 with KOH. The cell suspension was filtered, then centrifuged (440×g for 30 s) and resuspended in Kraftbruhe solution.

### Patch-clamp Recordings in Myocytes

Single ventricular myocytes were settled to a recording chamber on the inverted microscope (Olympus IX-70, Japan). Whole-cell patch-clamp recording was performed with an Axopatch 200B amplifier (Axon Instruments Inc., Union City, CA, USA), as described before [12], [14], [16]. Standard patch-clamp electrodes had a tip resistance of 2–4 MΩ when filled with pipette solution (in mM): 20 KCl, 110 potassium asparate, 1 MgCl_2_, 5 Na_2_-ATP, 10 EGTA, and 10 HEPES, pH adjusted to 7.2 with KOH. Voltage clamp protocols were controlled via PC using pClamp9.2 (Axon Instruments, USA) acquisition. Data were analyzed with pCamp software and plotted as current–voltage curves.

### Western Blot Analysis

Total protein was extracted from rat left ventricular wall and primary cultured neonatal cardiomyocytes, with the procedures essentially the same as described previously by our laboratory [12], [17]. Cardiac tissues or cardiomyocytes were lysed with standard lysis buffer on ice for 30 min and then centrifuged, finally the supernatant was collected. Samples were boiled for 5 min before fractionating by SDS-PAGE (10%–15% polyacrylamide gels). After transferred to PVDF membrane (Millipore, Bedford, MA), the membrane were blocked with blocking buffer (5% nonfat milk in PBS) at RT for 2 h. The membranes were incubated with primary antibodies to PLM (Abcam), Kir2.1(Santa Cruz Biotechnology Inc. USA), β-actin (Kangcheng Inc.) and GAPDH (Santa Cruz Biotechnology Inc. USA) at 4°C overnight. After carefully washed, the membrane was incubated with a secondary polyclonal antibody (Alexa Fluor, purchased from Santa Cruz Biotechnology) for 1 h at RT. Immunoreactive bands were captured and measured by the Odyssey System (LI-COR Bioscience, Lincoln, U.S.A.) and Odyssey v1.2 software. GAPDH or β-actin was chosen as an internal control.

### Quantification of miRNA Levels by Real-time PCR

Total RNA samples were isolated with mirVana™ miRNA Isolation Kit (Ambion, USA) from rat hearts. miR-151-5p level was measured using the mirVana™ qRT-PCR miRNA Detection Kit (Ambion, USA) with SYBR Green I following the manufacturer’s instructions. Real-time PCR was performed on a GeneAmp 5700 thermocycler for 40 cycles. Differences in expression of miR-151-5p between different RNA samples were calculated after normalization to U6. Primers were shown in Tab. 1.

**Table 1:** Comparison of cumulative arrhythmic durations, arrhythmia scores, incidence of ventricular fibrillation and mortalities in Ctl, MI, OVX and OVX+MI Groups.

Group	Ctl	MI	OVX	OVX+MI
Duration of PVB (s)	0	29.4±6.6*	0	127.8±12.6#
Duration of VT (s)	0	24.2±8.0*	0	121.9±17.9#
Duration of VF (s)	0	10.2±3.9*	0	94.4±16.0#
Score of arrhythmia	0	3.5±0.7*	0	4.9±0.1#
Incidence of VF	0	57.9%*	0	100.0%#
Motality	0	36.8%*	0	63.2%#

Data are mean ± SEM. There were nineteen animals in each group,

* P<0.05 compared with Ctl rats,

# P<0.05 compared with MI rats.

PVB, premature ventricular beats; VT, ventricular tachycardia; VF, ventricular fibrillation.

### Luciferase Assay

The luciferase assay was very similar to that described previously by our laboratory [18]. First we subcloned the 3′-untranslated region (3′-UTR) of *FXYD1* by PCR. Then the constructs were inserted into the multiple cloning sites (HindIII and SacI sites) downstream the luciferase gene in the pMIR-REPORT™ luciferase miRNA expression reporter vector (Ambion, Inc.), with the methods described previously [18], [19]. HEK293 cells (1×10^5^/well) were co-transfected with 1 µg PGL3–target DNA (firefly luciferase vector) and 20ng PRL-TK (TK-driven Renilla luciferase expression vector) with miR-151-5p mimics (NC, miR-151-5p+AMO-151-5p or AMO-151-5p, respectively) by lipofectamine™ 2000 according to the manufacturer’s instructions. Luciferase activities were measured 48 h after transfection with a dual luciferase reporter assay kit (Promega) on a luminometer (Promega) [18], [19].

### HEK293 Cell Culture

HEK293 cells (purchased from American Type Culture Collection, ATCC, Manassas, VA) were cultured in Dulbecco’s Modified Eagle Medium (DMEM) [20] with 10% fetal bovine serum and 100 µg/ml penicillin/streptomycin.

### Preparation of Primary Cultured Neonatal Cardiomyocytes

Primary cultured neonatal rat ventricular cardiomyocytes were isolated as described previously [18], [21]. Briefly, we cut rats’ (1–3 days old) chest wall and removed heart aseptically. Ventricle tissues were dissected, minced, and trypsinized at 37°C for 10 minutes, and this procedure was repeated for 4 times. After 2 hours of pre-plated, the non-adherent cardiomyocytes were removed and plated in 24-well plates in DMEM medium (Invitrogen) containing 10% FBS and 0.1 mM bromodeoxyuridine (Brdu, Sigma) for further experiments.

### Synthesis of miRNAs and miRNA Inhibitor

rno-miR-151-5p (MIMAT0000613) (sense: 5′-UCGAGGAGCUCACAGUCUAGUAU-3′; antisense: 5′-ACUAGACUGUGAGCUCCUCGAAU-3′) and its antisense inhibitor AMO-151-5p were synthesized by GenePharma (Shanghai, China). The sequence of AMO-151-5p is the exact antisense of the mature miRNA sequence (for rat: 5′-ACTAGACTGTGAGCTCCTCGA-3′). Additionally, a scrambled RNA was used as negative control (NC); sense: 5′-UUCUCCGAACGUGUCACGUAA-3′ and antisense: 5′-ACGUGACACGUUCGGAGAAUU-3′.

### Measurement of Intracellular Calcium Concentration ([Ca^2+^]_i_)

[Ca^2+^]_i_ fluorescence measurement in cardiomyocytes has been described previously [13], [22]. The peri-ischemic zone ventricular myocytes were derived from MI group and OVX+MI group. The same part of ventricular myocytes was derived from Ctl group and OVX group. Primary cultured rat neonatal cardiamyocytes were transfected with miR-151-5p, AMO-151-5p, miR-151-5p+AMO-151-5p and NC using lipofectamine^TM^2000. These myocytes were rinsed with Tyrode’s solution and then incubated with a Tyrode’s solution containing Fluo-3/AM (5µM) and Pluronic F-127 (0.03%) at 37°C for 45 min. The fluorescent density was detected by confocal laser scanning microscope (Fluoview-FV300, Olympus, Japan) using an excitation and emission wavelengths of 488 nm and 530nm to record resting calcium, respectively. Fluorescent intensities were recorded before (FI_0_) and after (FI) administration of KCl (60mM). The change of [Ca^2+^]_i_ was represented with ratio of fluorescence intensity (FI/FI_0_).

### Immunofluorescent

Hearts from rats were rapidly removed. After washed in ice-cold Tyrode solution, the samples were frozen in liquid nitrogen. Left ventricular apexes were continuously cut into 14 µm sections at −20°C with a cryostat (model CM19000, Leica), mounted onto slides. The tissue sections were fixed with 4% paraformaldehyde (pH 7.35 in PBS) at RT for 30 min, and then washed three times in PBS. We incubated the samples with Triton X-100 (0.4% in PBS) for 5 min to permeabilize the cell membrane, followed by blocking with 1% bovine serum albumin for 1.5 h at RT. Samples were incubated with goat polyclonal primary antibody to Kir2.1 (diluted 1∶100, Santa Cruz Biotechnology) and rabbit polyclonal primary antibody to PLM (diluted 1∶100, Abcam) on coverslips at 4°C overnight. Alexa Fluor 488-conjugated donkey anti-goat secondary antibody was used as secondary antibody (1∶2000 dilution). After the blocking procedure with 1% BSA, the cell membrane was stained with Alexa Fluor 594-conjugated donkey anti-rabbit secondary antibody (1∶2000 dilution) for 2 h. The sample was detected by confocal laser scanning microscope.

### co-Immunoprecipitation

Pre-cleared proteins from whole cell lysates were incubated with goat anti-Kir2.1 antibody or goat IgG which is conjugated to Protein A/G dynabeads (Santa Cruz) overnight at 4°C. After washing with 1% TBST, the co-IP targets were disassociated from the immobilized antibodies on the Protein A/G dynabeads by the gentle (non-reducing, non-denaturing) elution buffer. Eluted proteins were separated using 15% SDS-PAGE. Western blot was used to detect the expression of PLM with a rabbit primary antibody and then reacted with the second antibody (Alexa Fluor). Immunoreactive bands were captured by the Odyssey System (LI-COR Bioscience, Lincoln, U.S.A.).

### In vivo Gene Transfection

This method has been described previously [18]. Rats were divided into 2 groups randomly (NC group and AMO group) and anesthetized with sodium pentobarbital (40 mg/kg) intraperitoneal (i.p.). A 1.5 cm incision was cut along the left side of the sternum and dissected the chest wall bluntly and aseptic. AMO-151-5p (80 µg) and negative control (NC, 80 µg) were pretreated with lipofectamine™ 2000 (Invitrogen) and were injected into myocardium with a 26-gauge needle respectively. Intramuscular injections were made in approximately 10 sites. The standard lead II electrocardiogram was continuously recorded 6 hours after transfection.

### Data Analysis

All data are presented as mean ± SEM. T-test is used for two groups comparison. Compare incidence of arrhythmia and mortality among different groups were made by *χ^2^*-test. One-way analysis of variance (ANOVA) was used to compare data when more than two groups were examined. P<0.05 was considered to show a statistical difference significantly.

## Results

### Estrogen Deficiency Exacerbates Ischemic Arrhythmias and Mortality

Rats were subjected to induction of ventricular arrhythmias in response to acute myocardial ischemia (MI) following coronary artery ligation. Electrical disorders were observed for continuous 1 h after MI. The incidence and duration of ventricular arrhythmias were calculated. Arrhythmias of ventricular tachycardia (VT) and reversible ventricular fibrillation (VF) were observed both in MI and OVX+MI rats. Moreover, the ischemic arrhythmias occurred more often in OVX+MI group than MI group (n = 19, P<0.05, Table 1). Cumulative arrhythmic durations and arrhythmia scores were significantly higher in OVX+MI group than that in MI group (n = 19, P<0.05, Table 1). By comparison, arrhythmias were hardly observed in OVX group and Ctl group without MI.

We further assess the mobility rates of rats from different groups. Results showed that the mortality rate was higher in MI (36.8%; n = 19, P<0.05, *χ^2^*-test) than in Ctl group (0%). Moreover, it was 71.7% higher in OVX +MI (63.2%) than that in MI rats (36.8%).

### Estrogen Deficiency Exacerbates MI-induced Increases in Cardiac [Ca^2+^]_i_

**Figure 1:**
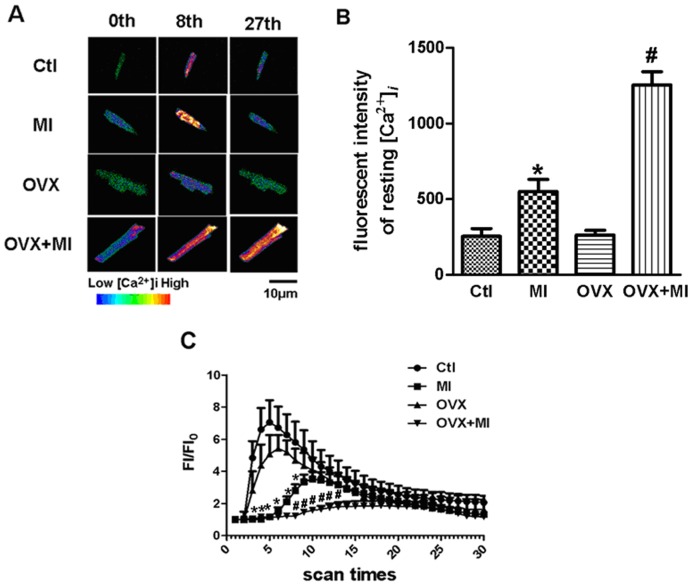
Intracellular Ca^2+^ of cardiomyocytes was observed with confocal laser scanning microscope. (A) Thirty fluorescence images of single cardiomyocyte were scanned at the interval of 10 s. 0, 8th, 27th second images are shown from Ctl, MI, OVX and OVX+MI group, respectively. (B) Resting [Ca^2+^]i values of each group. (C) A linear graph showed comparison of all groups. These linear graphs showed the difference in FI/FI0 among Ctl group (n* = *11), MI group (n = 10), OVX group (n = 16) and OVX+MI group (n = 10). *P<0.05 vs. Ctl group, ^#^P<0.05 vs. MI group.

During early myocardial ischemia, intracellular calcium overload has been considered as an underlying mechanism of cardiac injury. Compared with MI group, ([Ca^2+^]_i_) in myocytes from OVX+MI group was significantly higher (n = 10 in both OVX+MI and MI group, P<0.05, Fig. 1A, B). While the sensitivity to KCl stimulation was significantly lower in OVX+MI compared with MI group (n = 10 in both OVX+MI and MI group, P<0.05, Fig. 1C). The time to peak of FI/FI0 was prolonged in OVX+MI myocytes compared with that in MI group (n = 10 in both OVX+MI and MI group, P<0.05, Fig. 1C). But there was no significant difference in the time to peak and amplitude of FI/FI0 between Ctl group and OVX group.

### Estrogen Deficiency Exacerbates MI-induced Decreases in Electrophysiology

**Figure 2:**
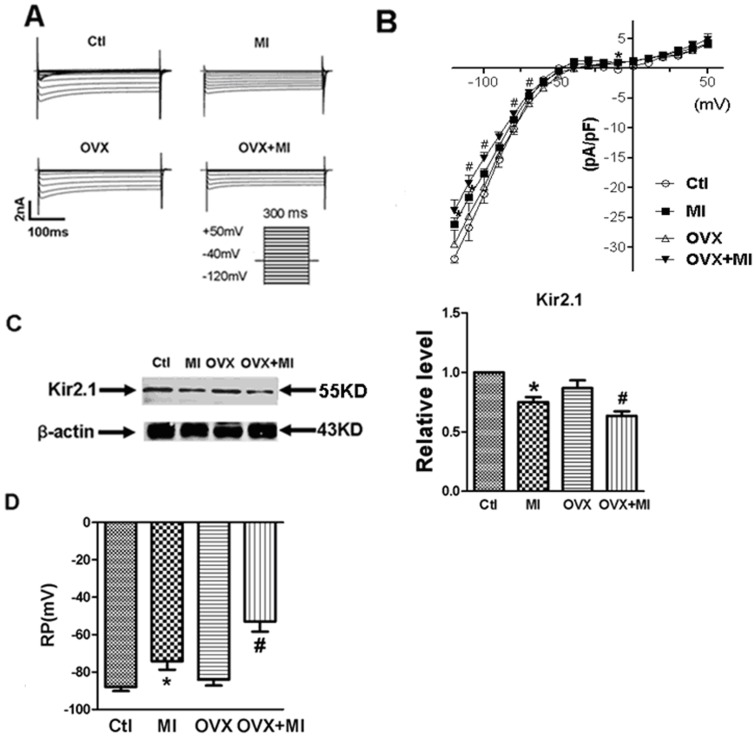
Changes of *I*_K1_, Kir2.1 expression and resting membrane potential in ventricular myocytes from Ctl, MI, OVX and OVX+MI group. (A) Representative of *I*_K1_ from different groups. (B) *I*–*V* relation curve of *I*_K1_ and the stimulation protocol. Mean data from both MI (n = 11) and OVX+MI (n = 10) group are significantly different from Ctl group (n = 11) and MI (n = 11), respectively. *P*<*0.05 vs. Ctl group, ^#^P*<*0.05 vs. MI group. (C) Acute myocardial infarction on Kir2.1 protein expression. Left: Kir2.1 protein levels from 4 group animals (n = 7), data were normalized to β-actin. Right: representative image for Kir2.1 and β-actin. (D) Resting membrane potential (RP; mean ± SEM) from tissue strips dissected from Ctl hearts (n = 5), MI (n = 6), OVX (n = 6) and OVX+MI hearts (n = 7) 1 h after coronary artery ligation. *P<0.05 MI vs. Ctl; ^#^P<0.05 OVX+MI vs. MI; unpaired Student’s *t*-test.

There was no significant difference in *I_K1_* between Ctl group and MI group. In line with this observation, Kir2.1 (the major subunit of cardiac inward rectifier K^+^ channel carrying *I*_K1_ current) level was not significantly different observed between OVX and Ctl groups, too. On the other side, *I*_K1_ current densities in ventricular cardiomyocytes from OVX+MI rats were decreased compared with MI rats (n = 11 in MI group, n = 10 in OVX+MI group, P<0.05, Fig. 2A, B). Coincidentally, Kir2.1 protein level was significantly down-regulated in OVX+MI rats compared to MI rats (n = 7, P<0.05, Fig. 2C). Moreover, the resting membrane potential was depolarized to a greater extend in OVX+MI than in MI hearts (Fig. 2D).

### Estrogen Deficiency Exacerbates MI-induced Upregulation of PLM Expression via Down-regulating miR-151-5p

**Figure 3:**
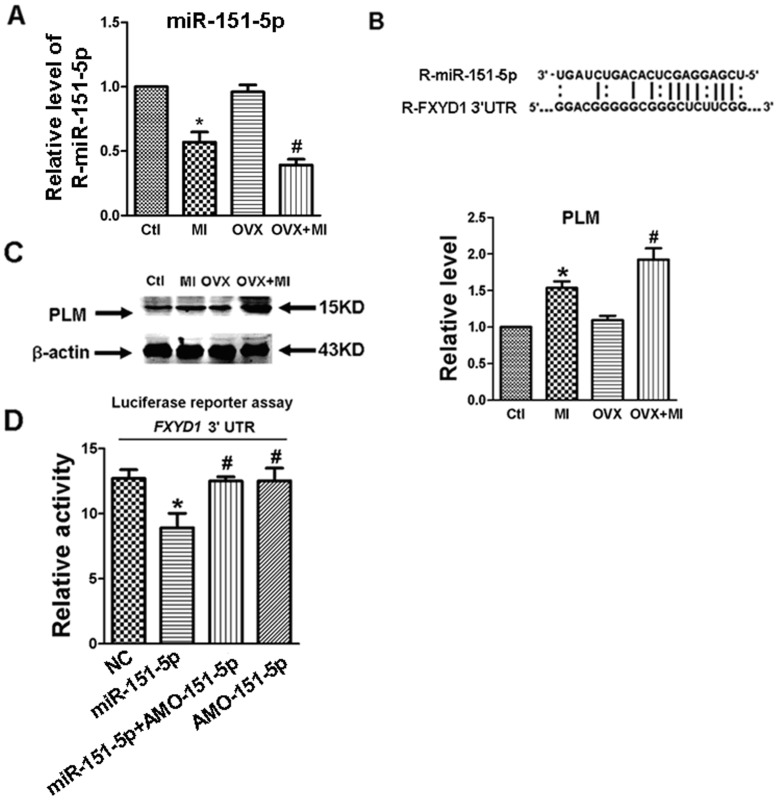
Estrogen deficiency exacerbates MI-induced upregulation of PLM expression via down-regulating miR-151-5p. (A) Relative expression of miR-151-5p in rat hearts in each group. (n = 5 hearts for each group). *P<0.05 vs. Ctl, ^#^P<0.05 vs. MI. (B) The complementary sequences between miR-151-5p and its putative Sites within the 3′UTRs of rat. (C) Effect of acute myocardial infarction on PLM protein expression. Left: representative image for PLM and β-actin. Right: PLM protein levels from 4 group animals (n = 8), data were normalized to β-actin. *P<0.05 MI vs. Ctl; ^#^P<0.05 OVX+MI vs. MI; unpaired Student’s *t*-test. (D) Verification of interactions between rat miR-151-5p and the 3′-UTRs of rat *FXYD1* in HEK293 cells, determined by luciferase reporter activity. HEK293 cells were transfected with miR-151-5p alone, miR-151-5p+AMO-151-5p, AMO-151-5p alone or a negative control (NC) with lipofectamine 2000. Luciferase activities were assayed after 48 hours of transfection. *P<0.05 vs. NC, unpaired Student’s *t*-test; ^#^P<0.05 vs. miR-151-5p alone, unpaired Student’s *t*-test; n = 3 independent batches of cells for each group.

To explore the possible involvement of miRNAs in the expression regulation of PLM, we first measured the expression levels of miR-151-5p which have the potential to regulate its target gene (*FXYD1*) expression. As illustrated in Fig. 3A, miR-151-5p was significantly down-regulated in ventricular cardiac tissues of OVX +MI rats compared with MI animals, though OVX alone did not alter miR-151-5p expression. Fig. 3B shown that miR-151-5p could bind to FXYD1 gene 3′-UTR based on computational prediction using the miRNA Microcosm Targets hosted by European Bioinformatics Institute [23], [24]. Watson-Crick complementarity is shown in bold and connected by “I”, however, the matched base “G” and “U” connected by “:”.

PLM expressed in the heart is known to be able to alter cardiac membrane excitability. In order to investigate whether PLM plays a role in the vulnerability to acute ischemia-induced ventricular arrhythmias and cardiac dysfunction in rats with long-term estrogen deficiency, we assessed the effects of acute MI on PLM. Consistent with the alternation of myocyte membrane excitability, western blot analysis revealed significant up-regulation of PLM at the protein level in cardiac tissue in OVX+MI rats compared to MI rats (n = 8, P<0.05, Fig. 3C), and this up-regulation was 1.5-fold greater in OVX+MI than in MI rats.

Previous studies have indicated that miRNA-binding sites are transferable and sufficient to confer miRNA-dependent gene silencing. The ability of miR-151-5p to repress *FXYD1* was verified by luciferase reporter activity assays in HEK293 cells that express minimal endogenous miR-151-5p (Fig. 3D). When the luciferase vector carrying the 3′-UTR of *FXYD1* was co-transfected with miR-151-5p, luciferase activity was robustly diminished, compared with transfection of a scrambled miRNA for negative control (NC). The inhibitory effect of miR-151-5p was antagonized by its antisense AMO-151-5p.

### miR-151-5p Represses PLM, Up-regulates Kir2.1 Expression and Decreases Intracellular Calcium Transient

**Figure 4:**
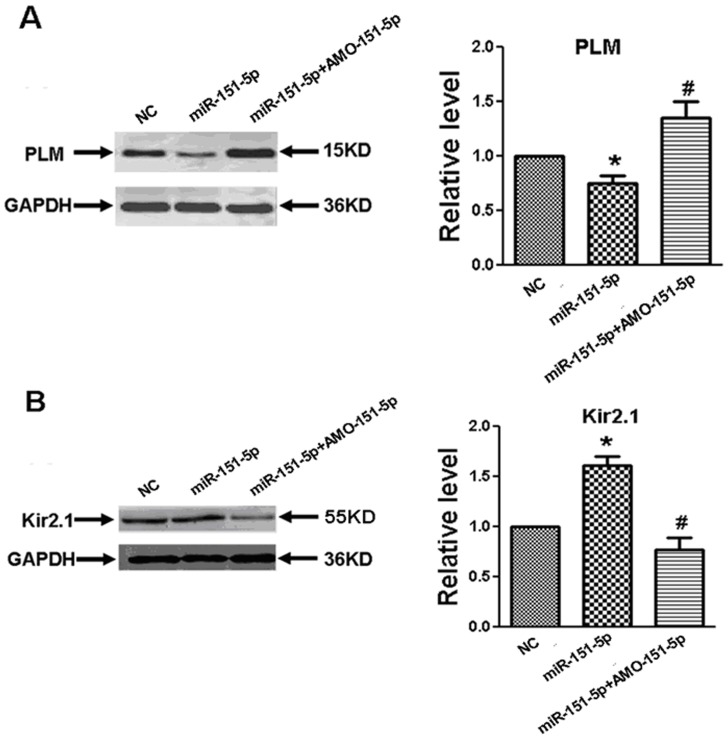
Effects of miR-151-5p on the expression of PLM (A) and Kir2.1 (B). The totle protein samples were isolated from neonatal rat ventricular myocytes after 48 hours of transfection with miR-151-5p alone, miR-151-5p+AMO-151-5p, or a negative control (NC). Data are expressed as mean ± SEM from five batches of cells. *P<0.05 vs. NC; unpaired Student’s *t*-test.

**Figure 5:**
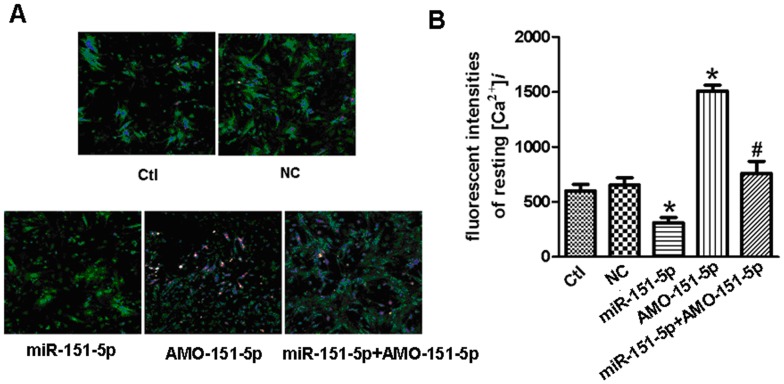
Effects of miR-151-5p on the levels of intracellular calcium in neonatal cardiomyocytes. miR-151-5p repressed the resting intracellular calcium, which was effectively restricted by AMO-151-5p. Data are expressed as mean ± SEM for 10-14 cells from at least three individual experiments. *P<0.05 vs. NC, ^#^P<0.05 vs. miR-151-5p.

We subsequently evaluated the ability of miR-151-5p to repress PLM and promote Kir2.1 expression in primary culture neonatal rat myocytes. As depicted in Fig. 4A, B, transfection of miR-151-5p remarkably reduced the protein level of PLM and increased the protein level of Kir2.1 and co-transfection of AMO-151-5p abolished the changes induced by miR-151-5p (Fig. 4A, B). Cardiac arrhythmias can be triggered by intracellular Ca^2+^ overload. We observed here that miR-151-5p markedly decreased the resting [Ca^2+^]_i_ in primary culture neonatal rat myocytes, and this decrease was abolished by AMO-151-5p (Fig. 5A, B).

### Correlation between Kir2.1 and PLM Expression in Rat Ventricular Tissue

**Figure 6:**
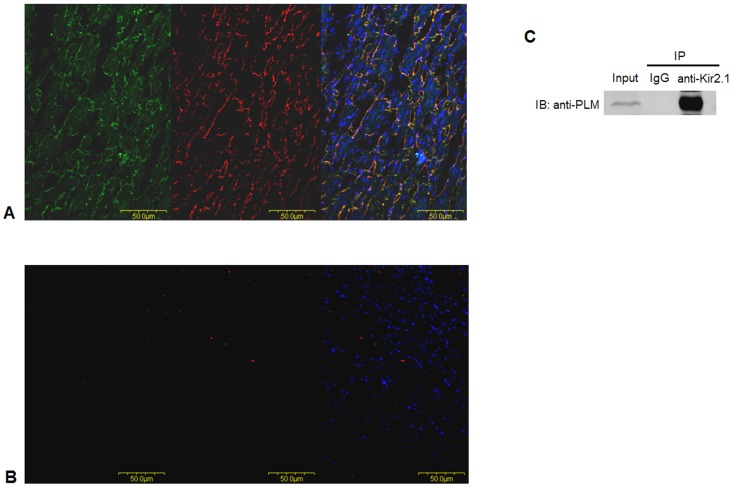
Co-localization of Kir2.1 and PLM in rat ventricular tissue. (A) Subcellular distribution of Kir2.1(green) and PLM (red) proteins from left ventricular slices is detected by immunofluorescent analysis. (B) Goat IgG (left) and rabbit IgG (middle) instead of Kir2.1 and PLM primary antibody were used to confirm the antibody specificity. (C) Immunoprecipitation was performed using anti-Kir2.1 antibody, precipitates were analyzed by western blot with anti-PLM antibody (IP = immunoprecipitation; IB = immunoblotting).

The above results suggest that PLM may interact with Kir2.1. We studied the subcellular localizations of Kir2.1 and PLM proteins using immunofluorescent with double staining for Kir2.1 (green) and PLM (red). Under a confocal microscope, both Kir2.1 and PLM proteins were stained along the cytoplasmic membrane (Fig. 6A). In order to confirm the antibody specificity, we used goat IgG (left) and rabbit IgG (middle) instead of Kir2.1 and PLM primary antibody in Figure 6B. There is no significant fluorescence signal in rat ventricular tissue but only stained nuclei with DAPI. So we confirmed that our immunofluorescent staining is specific and Kir2.1 and PLM co-localize on cardiomyocyte membrane. To further confirm their correlation, we detect their interaction with co-immunoprecipitation. We detected PLM expression in precipitated immune complex to identify that Kir2.1 and PLM co-localized on cardiomyocyte membrane and these two proteins interact directly (Fig. 6C).

## Discussion

We demonstrate here that estrogen deficiency can exacerbate cardiac malfunction and promote ventricular arrhythmias induced by acute myocardial ischemia in rats. This arrhythmia-promoting effect of estrogen deficiency was accompanied by disorders of cellular electrophysiology including suppression of *I*_K1_ and intracellular calcium overload. Moreover, estrogen deficiency exacerbates acute MI-induced down-regulation of miR-151-5p, which may underlie the up-regulation of PLM protein level and down-regulation of Kir2.1 potassium channel expression. Our study therefore suggests that a mechanism by which estrogen deficiency promotes ischemic arrhythmias may be mediated through the miR-151-5p–PLM–ion channel signaling pathway.

Phospholemman (PLM) is a tissue-specific regulator of the Na^+^-K^+^-ATPase, which belongs to the FXYD family of proteins [25]. Sarcolemmal PLM (*FXYD1*) is phosphorylated by PKC and PKA in the heart [26], [27]. Na^+^-K^+^ pump activity was regulated by PLM in a way analogous to the regulation of SERCA by phospholamban. Na^+^-K^+^ pump was inhibited by un-phosphorylated PLM, while this inhibition and stimulates pump activity was relieved phosphorylated PLM [28].

Evidence exists from clinical observations indicating that cardiac diseases including ventricular arrhythmia morbidity and mortality increase in post-menopausal women. However, it can’t be explained by inhibition of atherosclerosis before atherosclerotic plaque formation. So the potential mechanisms by which estrogen deficiency increases propensity of ventricular arrhythmia remained unclear.

An important finding in this study is that although estrogen deficiency alone does not alter cardiac electrophysiology and miR-151-5p expression, it indeed worsened the electrical disturbances in the setting of acute MI.

miRNAs might play critical roles in the pathophysiology of AMI, which have been suggested by several recent reports [29], [30], [31], [32]. Dramatical down-regulation of miR-29 was observed in the region of the fibrotic scar after MI [31]. MiR-21 was reported to play a protective role in MI [29]. More importantly, our previous study revealed that miR-1 was a negative regulator of connexin 43 and Kir2.1 expression in rats with MI [18]. In addition, it is likely that multiple miRNAs contribute to controlling arrhythmogenicity of the heart and that different miRNAs are involved in different types of arrhythmias under different pathological conditions of the heart. The present study revealed, for the first time, the role of miRNA in ischemic ventricular arrhythmias in rats with estrogen deficiency and miR-151-5p as a new arrhythmogenic miRNA acting on *FXYD1*. We first found that miR-151-5p was significantly down-regulated in ventricular cardiac tissues of OVX +MI rats compared with MI rats, though OVX alone did not alter miR-151-5p expression.

We further researched on the expression of PLM, which was encoded by *FXYD1*, in the heart. Computational prediction has shown that miR-151-5p has potential to bind to FXYD1 gene 3′-UTR. In the heart, PLM directly interacts [33] with Na^+^-K^+^-ATPase, Na^+^/Ca^2+^ exchanger [34], [35], [36] and L-type Ca^2+^ channel [37]. Both Na^+^-K^+^-ATPase [38], [39] and Na^+^/Ca^2+^ exchanger [34], [40], [41] were repressed by PLM in cardiac myocytes. In heterologous expression systems, the gating of cardiac L-type Ca^2+^ channel and increases Ca^2+^ influx were regulated by PLM during the repolarization phase of the cardiac action potential [37]. Consequently, PLM plays an important role in regulation of excitation-contraction (EC) coupling by modulating intracellular Na^+^ and Ca^2+^ homeostasis. The risks of arrhythmogenesis may be maximized via upregulated PLM, which can increase Na^+^ overload and Ca^2+^ overload by inhibiting Na^+^-K^+^-ATPase and Na^+^/Ca^2+^ exchanger. Arrhythmias and contractile dysfunctions may be induced by [Ca^2+^]_i_ overload.

Sehl *et al* firstly report that *FXYD1* gene expression level was upregulated after MI in the rat [42]. Zhang *et al* also verified that PLM protein expression levels in rats was upregulated by 2.4-and 4-fold at 3 and 7 days post-MI, respectively [43]. We found here that PLM protein level was up-regulated in MI and OVX+MI rat hearts, which was in accordance with previous studies. Our data also present the first demonstration that miR-151-5p can inhibits the expression of its target gene (*FXYD1,* Fig. 4a). Based on previous studies and the present data, it is speculated that miR-151-5p down-regulation may result in reduction of Kir2.1/I_K1_, RP, Na^+^-K^+^-ATPase [38], [39] and Na^+^-Ca^2+^ exchanger [34], [40], [41], leading to calcium and sodium overload in ventricular myocytes. These downstream alterations may be a mechanism underlying the ventricular arrhythmogenic potential of down-regulated miR-151-5p. Moreover, we detected the co-relationship between Kir2.1 and PLM expression in rat ventricular tissue. We first found that Kir2.1 is co-precipitated with PLM in rat ventricular tissue.

Finally, the ability of the AMO-151-5p to cause calcium overload suggests the potential of miR-151-5p mimic as a drug for molecular conversion of ventricular arrhythmia to sinus rhythm. Normalization of miR-151-5p level to the normal range may well be a novel strategy for arrhythmic therapy in the clinical setting. In this sense, miR-mimics approach appears to represent a wise choice.

It is worth mentioning that miR-151-5p has been implicated in many other pathological conditions. For example, recent studies have shown that miR-151 is correlated with hepatocellular carcinoma cell migration and invasion [44]. These findings indicate the widespread pathophysiological function of miR-151-5p in humans. Moreover, in all these cases, abnormal regulation of miR-151-5p is a common finding; therefore, keep balance of miR-151-5p expression may be a useful strategy for many diseases associated with miR-151-5p.

It should be noted that although our study identified miR-151-5p as an important factor in increasing susceptibility of MI-induced ventricular arrhythmias in rats with estrogen deficiency, it does not exclude other mechanisms for determining susceptibility of MI-induced ventricular arrhythmias, and our findings in experimental models may not be applied directly to human ventricular arrhythmias. Nonetheless, our study does provide novel insight into experimental ventricular arrhythmias and a new aspect of the role of miRNAs in cardiac disease. Although miR-151-5p is involved in increasing susceptibility of MI-induced ventricular arrhythmias in rats with estrogen deficiency, whether it also plays a role in ventricular arrhythmia of other causes is unclear.

In order to verify the effect of miR-151-5p knockdown on the increased ventricular arrhythmias vulnerability, we used in vivo gene transfer technique to transfect AMO-151-5p into myocardium. We found that the duration of PVB prolonged after knockdown the expression of miR-151-5p with AMO-151-5p (Fig. S1A). But the low mortality didn’t change in both NC and AMO-151-5p group. So we ligated coronary artery to construct myocardial ischemia model after in vivo gene transfection. We found that the mortality increased significantly in AMO-151-5p+MI group (Fig. S1B). In conclusion, the increased ventricular arrhythmias vulnerability in response to acute ischemia in rats with estrogen deficiency is critically dependent upon down-regulation of miR-151-5p compared with MI heart only. These results indicated that estrogen deficiency exacerbated cardiac diastolic function (reflected by LVEDP) and systolic function (reflected by dP/dt which was affected by the decreased heart rate) in MI hearts (Tab. S2) not only by reducing *I*_K1_ in MI cardiomyocytes, and by overloading [Ca^2+^]_i_ as well.

The new insights that we have obtained from this study will help in the development of safer and more effective drugs. Knowledge of miRNAs from this work may provide insight into new drugs target for estrogen deficiency and MI complication treatment. These findings support the proposal that miR-151-5p could be a potential therapeutic target for the prevention of ischemic arrhythmias in post-menopausal women.

## Supporting Information

Figure S1 **Effect of miR-151-5p knockout on the increased ventricular arrhythmias vulnerability in vivo.** (A) In vivo gene transfer technique was used to transfect AMO-151-5p into myocardium with lipofectamine™ 2000. ECG was recorded after 6h transfection. Data are mean ± SEM. There were seven animals in NC group and nine animals in AMO-151-5p group, *P<0.05 compared with NC group. PVB, premature ventricular beats. (B) Mortality was calculated after ligating coronary artery with in vivo gene transfection.
(TIF)

Table S1 **Reverse transcription specific primers, forward and reverse primers sequence for miRNA real-time PCR.**
(DOC)

Tables S2 **Comparisons of Hemodynamic Parameters of Ctl, MI, OVX and OVX+MI rats.**
(DOC)
