# Supplementary figures and images for: Downregulation of miR-151-5p Contributes to Increased Susceptibility to Arrhythmogenesis during Myocardial Infarction with Estrogen Deprivation
Source: PLoS One. 2013 Sep 9;8(9):e72985. doi: 10.1371/journal.pone.0072985 (PMC3767733; doi:10.1371/journal.pone.0072985)

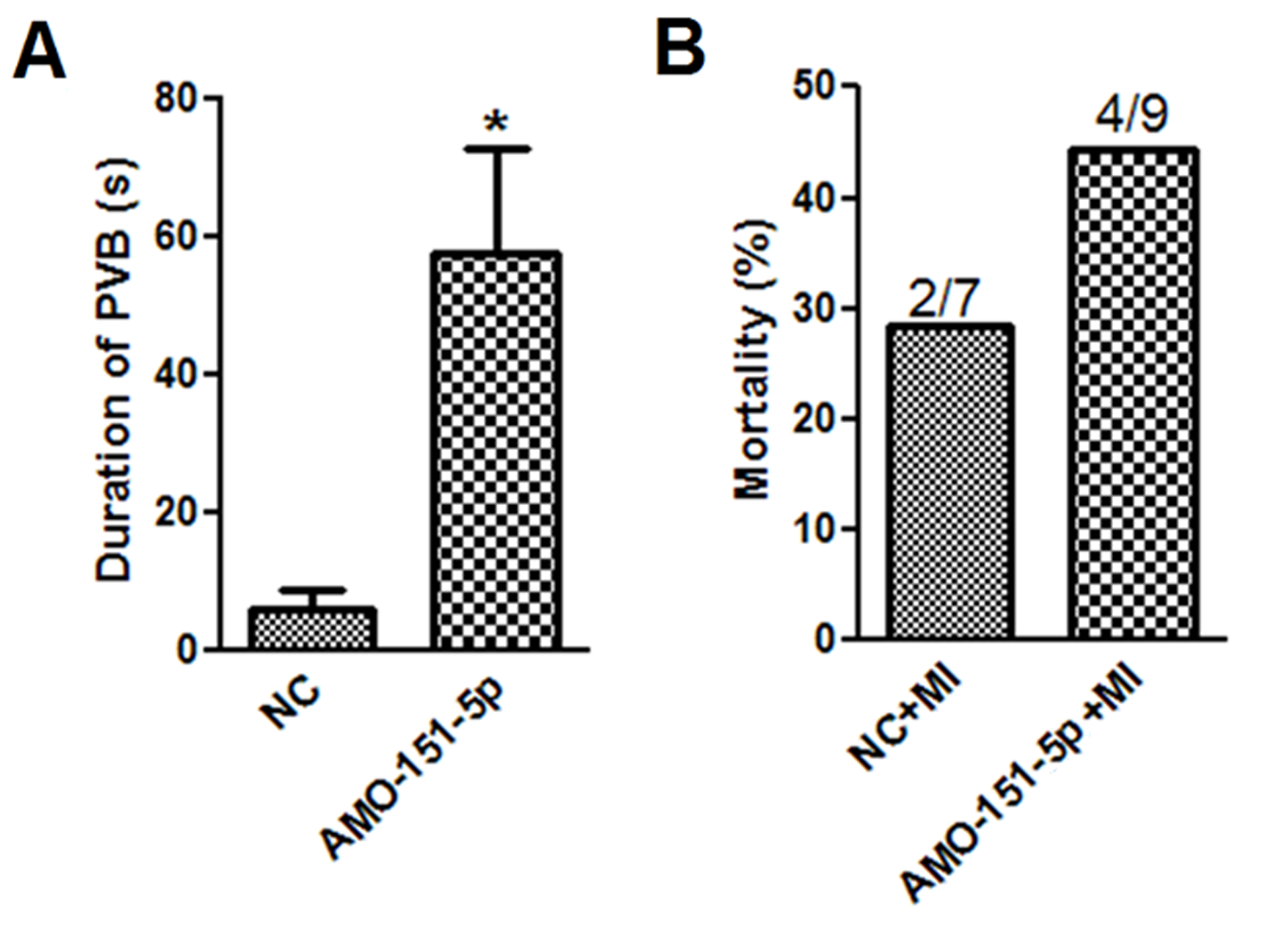

Supplement: Figure S1 — Effect of miR-151-5p knockout on the increased ventricular arrhythmias vulnerability in vivo. (A) In vivo gene transfer technique was used to transfect AMO-151-5p into myocardium with lipofectamine™ 2000. ECG was recorded after 6h transfection. Data are mean ± SEM. There were seven animals in NC group and nine animals in AMO-151-5p group, *P<0.05 compared with NC group. PVB, premature ventricular beats. (B) Mortality was calculated after ligating coronary artery with in vivo gene transfection. (TIF) [file pone.0072985.s001.tif]
