# Supplementary material for: Downregulation of miR-151-5p Contributes to Increased Susceptibility to Arrhythmogenesis during Myocardial Infarction with Estrogen Deprivation
Source: PLoS One. 2013 Sep 9;8(9):e72985. doi: 10.1371/journal.pone.0072985 (PMC3767733; doi:10.1371/journal.pone.0072985)
Supplement: Table S1 — Reverse transcription specific primers, forward and reverse primers sequence for miRNA real-time PCR. (DOC) [file pone.0072985.s002.doc]

**Table S1. Reverse transcription specific primers, forward and reverse primers sequence for miRNA real-time PCR**

| miRNAs | RT Primer 5’ to 3’ and forward and reverse Primer 5’ to 3’ |
| --- | --- |
| rno-miR-151-5p | RT:GTCGTATCCAGTGCGTGTCGTGGAGTCGGCAATTGCACTGGATACGACACTAGA; F:TCGAGGAGCTCACAG; R:CAGTGCGTGTCGTGGAGT |
| U6 | RT:CGCTTCACGAATTTGCGTGTCAT; F:GCTTCGGCAGCACATATACTAAAAT; R:CGCTTCACGAATTTGCGTGTCAT |

RT, reverse transcription specific primers; F, forward primers; R, reverse primers.
