# Supplementary material for: Downregulation of miR-151-5p Contributes to Increased Susceptibility to Arrhythmogenesis during Myocardial Infarction with Estrogen Deprivation
Source: PLoS One. 2013 Sep 9;8(9):e72985. doi: 10.1371/journal.pone.0072985 (PMC3767733; doi:10.1371/journal.pone.0072985)
Supplement: Tables S2 — Comparisons of Hemodynamic Parameters of Ctl, MI, OVX and OVX+MI rats. (DOC) [file pone.0072985.s003.doc]

**Table S2. Comparisons of Hemodynamic Parameters of Ctl, MI, OVX and OVX+MI rats**

| Group | Ctl | MI | OVX | OVX+MI |
| --- | --- | --- | --- | --- |
| HR (beats/min) | 411.2 ± 8.1 | 355.1 ± 22.3* | 395.7 ± 18.0 | 294.6 ± 15.3^#^ |
| LVSP (mmHg) | 172.2 ± 8.8 | 109.5 ± 8.7* | 140.6 ± 13.3 | 69.2 ± 6.3^#^ |
| LVEDP (mmHg) | 2.9 ± 1.1 | 10.1 ± 0.9* | 2.3 ± 0.5 | 13.6 ± 1.1^#^ |
| + dP/dtmax (mmHg/s) | 7082.2 ± 508.5 | 5450.0 ± 500.3* | 6996.7 ± 383.4 | 3331.1 ± 398.7^#^ |
| - dP/dtmax(mmHg/s) | 5398.9 ± 375.2 | 3833.3 ± 548.8* | 5578.9 ± 527.2 | 2406.7 ± 297.6^#^ |

Data are the mean ± SEM. There were nine animals in each group, *P < 0.05 compared with Ctl rats, ^#^P < 0.05 compared with MI rats. estrogen-deficiency, ovariectomy；MI，myocardial infarction; HR, heart rate; LVSP, left ventricle systolic pressure; LVEDP, LV end-diastolic pressure; dP/dtmax, maximum first differentiation of LV pressure.
